# Aspirin and Simvastatin Combination for Cardiovascular Events Prevention Trial in Diabetes (ACCEPT-D): design of a randomized study of the efficacy of low-dose aspirin in the prevention of cardiovascular events in subjects with diabetes mellitus treated with statins

**DOI:** 10.1186/1745-6215-8-21

**Published:** 2007-08-28

**Authors:** Giorgia De Berardis, Michele Sacco, Virgilio Evangelista, Alessandro Filippi, Carlo B Giorda, Gianni Tognoni, Umberto Valentini, Antonio Nicolucci, ACCEPT-D Study Group

**Affiliations:** 1 Department of Clinical Pharmacology and Epidemiology, Consorzio Mario Negri Sud, Santa Maria Imbaro, Italy; 2 Department of Translational Pharmacology, Consorzio Mario Negri Sud, Santa Maria Imbaro, Italy; 3 Metabolism and Diabetes Unit, Chieri, Italy; 4 Internal Medicine, Department of Medical and Surgical Sciences, University of Brescia, Italy; 5 Italian College of General Practitioners (SIMG), Florence, Italy

## Abstract

**Background:**

Despite the high cardiovascular risk, evidence of efficacy of preventive strategies in individuals with diabetes is scant. In particular, recommendations on the use of aspirin in patients with diabetes mostly reflect an extrapolation from data deriving from other high risk populations. Furthermore, the putative additive effects of aspirin and statins in diabetes remain to be investigated. This aspect is of particular interest in the light of the existing debate regarding the need of multiple interventions to reduce total cardiovascular risk, which has also led to the proposal of a polypill. Aim of the study is to evaluate the efficacy of aspirin in the primary prevention of major cardiovascular events in diabetic patients candidate for treatment with statins. These preventive strategies will be evaluated on the top of the other strategies aimed at optimizing the care of diabetic patients in terms of metabolic control and control of the other cardiovascular risk factors.

**Methods/Design:**

The ACCEPT-D is an open-label trial assessing whether 100 mg/daily of aspirin prevent cardiovascular events in patients without clinically manifest vascular disease and treated with simvastatin (starting dose 20 mg/die). Eligible patients will be randomly assigned to receive aspirin + simvastatin or simvastatin alone. Eligibility criteria: male and female individuals aged >=50 years with diagnosis of type 1 or type 2 diabetes, already on treatment with statins or candidate to start the treatment (LDL-cholesterol >=100 mg/dL persisting after 3 months of dietary advise). The primary combined end-point will include cardiovascular death, non-fatal myocardial infarction, non-fatal stroke, and hospital admission for cardiovascular causes (acute coronary syndrome, transient ischemic attack, not planned revascularization procedures, peripheral vascular disease). A total of 515 first events are needed to detect a reduction in the risk of major cardiovascular events of 25% (alpha = 0.05; 1-beta = 0.90). Overall, 5170 patients will be enrolled. The study will be conducted by diabetes specialists and general practitioners.

**Discussion:**

The study will provide important information regarding the preventive role of aspirin in diabetes when used on the top of the other strategies aimed to control cardiovascular risk factors.

**Trial registration:**

Current Controlled Trials ISRCTN48110081.

## Background

Macrovascular complications represent the leading cause of morbidity, mortality, and resource consumption in type 2 diabetes, and their burden is expected to grow in the next years due to the increasing incidence of the disease worldwide.

Individuals with type 2 diabetes have a two- to fourfold increased risk of cardiovascular disease (CVD) compared with non-diabetic subjects and CVD mortality rates are 1.5–4.5 times higher than in the general population [1]. The increase in the incidence of coronary events is greater for more severe clinical outcomes, such as myocardial infarction and sudden death, than for less serious outcomes, such as angina pectoris [1]. Furthermore, the case fatality rate after a myocardial infarction among individuals with diabetes is higher than that for patients without diabetes; after a first cardiac event, 50% of patients with diabetes die within one year, and half of those who die do so before they reach the hospital (sudden deaths) [2]. The high incidence of sudden deaths makes primary prevention particularly relevant in this population. In addition, it has been shown that diabetic patients without previous myocardial infarction have as high a risk of myocardial infarction as non-diabetic patients with previous myocardial infarction [3]. These findings make the differentiation between primary and secondary prevention in individuals with diabetes almost irrelevant and suggest the need for treating cardiovascular risk factors aggressively.

Despite the very high cardiovascular risk profile, evidence of efficacy of preventive strategies in individuals with diabetes is surprisingly scant. In particular, the existing recommendations [4] regarding the use of aspirin and statins for the prevention of cardiovascular events in patients with diabetes reflect more an extrapolation from data deriving from other high risk populations, rather than reliable evidence based on studies specifically designed for individuals with diabetes. The substantial lack of clear evidence is reflected by the low use of this drug in clinical practice; in fact, only 10% of diabetic patients are treated with aspirin for the prevention of cardiovascular events [5].

While the efficacy of aspirin versus placebo in patients with diabetes is currently under investigation, the putative additive effects of aspirin and statins in this population remain to be investigated. This aspect is of particular interest in the light of the existing debate regarding the need of multiple interventions to reduce total cardiovascular risk, which has also led to the proposal of a polypill [6]. To this respect, a recent nested case-control study suggests that the greater benefit in terms of mortality reduction in patients with ischemic heart disease is achieved when the multiple drug intervention included statins, aspirin and β-blockers and/or ACE-inhibitors [7]. Nevertheless, no specific information is available for patients with diabetes mellitus, for whom there is the suspicion that aspirin could be less effective [8], making additional research urgently needed.

This situation is particularly worrisome when considering that a tight metabolic control, while representing a cornerstone of diabetes care, is not per se sufficient to substantially decrease the cardiovascular risk. On the other hand, there is a substantial lack of reliable information about the benefits of primary prevention strategies aimed at reducing the overall risk associated with diabetes. For all these reasons, the evaluation of cardiovascular preventive strategies in patients with diabetes represents a priority in terms of public health.

### Rationale to test the efficacy of aspirin for the prevention of cardiovascular events in diabetes mellitus

Data on primary prevention of cardiovascular events in individuals with diabetes and no evidence of cardiovascular disease are scarce. The meta-analysis on the efficacy of antiplatelet therapy showed a clear benefit for the whole population of over 140.000 subjects (22% reduction in the risk of major cardiovascular [CV] events), but no statistically significant benefit was documented in the subgroup of about 5.000 diabetic patients (7% risk reduction) [9]. More recently, an analysis on more than 1000 patients with diabetes enrolled in the Primary Prevention Project (PPP) confirmed a non statistically significant reduction of 10% in the incidence of major CV events in individuals treated with aspirin as compared with controls [10].

Another puzzling result regarding the efficacy of aspirin in primary prevention comes from the recent data of Womens' Health Study [11] suggesting biological differences between men in women in their response to aspirin. This study showed that aspirin had no significant effect on the risk of myocardial infarction or cardiovascular death neither in women without diabetes, nor in those with diabetes.

The lack of adequate statistical power can represent the more obvious explanation for the failure in documenting a clear benefit of antiplatelet therapy in the primary prevention of cardiovascular events in subjects with diabetes.

On the other hand, diabetes could represent a special case of aspirin resistance [8], although no specific studies have, to our knowledge, fully explored this hypothesis. The reason for the apparently reduced aspirin efficacy in diabetic patients remains largely unclear, however both, intraplatelet mechanisms and extra-platelet inflammatory-thrombogenic factors may contribute to the complexity of aspirin failure in diabetic patients.

In high risk patients treated with aspirin, inadequate suppression of Cox-1 as documented by high levels of serum thromboxane-2 [12] or urinary thromboxane metabolites [13], may be associated with increased risk of recurrent cardiovascular events.

Moreover, in some cases the antiplatelet effects of aspirin can be overwhelmed by aspirin-insensitive mechanisms of platelet activation and thrombus formation, mainly related to an up-regulated vascular inflammatory reaction [8,14].

From this point of view, several studies have documented that diabetes is associated with elevated levels of C-reactive protein [15] and endothelial adhesion molecules, considered as markers of the endothelial inflammatory response [16,17]. Diabetes is also associated with a reduction in the production of, and sensitivity to nitric oxide and PGI2, two of the most important antiaggregants. Furthermore, an increased platelet sensitivity to aggregant agents has also been described [18].

### Rationale for association of aspirin and statins

It has been recently shown that platelet response to aspirin is linearly reduced with increasing cholesterol plasma levels. The presence of dyslipidemia, particularly common among diabetic patients, could thus be at least partially responsible for a lower efficacy of aspirin in this population [19]. The concomitant use of statins could thus restore the normal platelet sensitivity to aspirin by reducing cholesterol levels.

One additional reason to hypothesize a positive effect of statins in improving platelet response to aspirin is related to their anti-inflammatory properties. Several studies have shown that treatment with statins is associated with a decrease in the levels of C-reactive protein in just a few weeks [20,21]. Statins also decrease the activity of inflammatory cells [22]. Clinical and experimental studies have shown that statins can reduce endothelial dysfunction by increasing the production of nitric oxide (NO), decreasing the synthesis of endothelin-1 and inhibiting the LDL cholesterol oxidation [23]. This class of drugs has also been shown to increase atherosclerotic plaque stability by reducing macrophage cholesterol accumulation and production of metalloproteases [23]. Therefore, if an upregulated vascular inflammatory reaction is responsible for aspirin resistance, it is plausible to hypothesize that statins would also improve the clinical response to aspirin therapy.

On the other hand, if platelets of diabetic patients were normally inhibited by aspirin, but other thrombogenic stimuli not inhibited by aspirin overwhelm aspirin action, statins could exert a complementary action by inhibiting these mechanisms; in fact, statins may exert antithrombotic effects by interfering with platelet adhesion and aggregation, expression of tissue factor and plasminogen activator inhibitor-1 (PAI-1), fibrinogen concentration and blood viscosity [22].

Given these premises, it is of crucial importance to conduct a large-scale, pragmatic trial to evaluate the effectiveness of aspirin use in primary prevention of cardiovascular events in association with statins therapy when included in a strategy of global risk control.

The choice of simvastatin is based on economical considerations; in fact, Simvastatin is the only statin available in Italy as an equivalent product, thus implying substantially lower costs in comparison with brand products.

If the combination of aspirin and simvastatin will prove to be effective in reducing diabetes morbidity and mortality, this will also translate into a dramatic decrease in the costs for the Italian health care system.

## Methods

### General objective

Aim of this trial is to evaluate the efficacy of low-dose aspirin (100 mg/daily) in the prevention of major cardiovascular events in diabetic patients without clinically manifest vascular disease and candidate for treatment with statins. These preventive strategies will be evaluated on the top of the other strategies (including lifestyle interventions and pharmacological treatments) aimed at optimizing the care of diabetic patients in terms of metabolic control and control of the other cardiovascular risk factors (obesity, hypertension, and smoking).

#### Primary objectives

To assess the effects of low dose aspirin on the incidence of major vascular events (defined as a combined end-point of cardiovascular death, non-fatal myocardial infarction, non-fatal stroke, or inpatient or outpatient hospital admission for cardiovascular causes, including acute coronary syndrome, not planned revascularization procedures, peripheral vascular disease), in a wide range of people with type 1 or type 2 diabetes with no clinical evidence of vascular disease and receiving statins treatment.

#### Secondary objectives

Additional aspects to be considered will include total and cause specific mortality, venous thromboembolic episodes, major hemorrhagic episodes, and total number of hospitalizations for cardiovascular causes (myocardial infarction, stroke, acute coronary syndrome, not planned revascularization procedures, heart failure, transient ischemic attack, peripheral vascular disease, lower limb revascularization procedures).

The study will also describe the natural history of the disease under routine clinical practice conditions, particularly with reference to the degree of control of the other cardiovascular risk factors.

The coherence in the effects of low dose aspirin across different patients subgroups defined according to their baseline characteristics/risk (i.e. gender, age, type of diabetes, metabolic control, baseline cholesterol levels, presence of microvascular complications, treatment with other cardiovascular drugs) will also be assessed.

### Study design

This is a trial designed to assess whether 100 mg/daily of aspirin prevent cardiovascular events in diabetic patients without clinically manifest vascular disease and treated with simvastatin (starting dose 20 mg/die).

A prospective, randomized, open, blinded endpoint evaluation (PROBE) design will be adopted [24].

**Figure 1:**
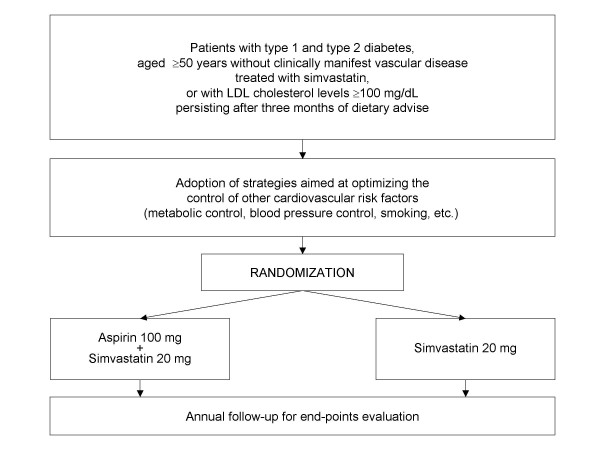
Study design.

Eligible patients will be randomly assigned (ratio 1:1) to receive aspirin + simvastatin or simvastatin alone (Figure 1).

### Study population

Male and female subjects aged ≥50 years are eligible for the study if they meet the following inclusion criteria:

#### Inclusion criteria

• Written informed consent to participate in this study;

• Clinical diagnosis of type 1 or type 2 diabetes, based on standard diagnostic criteria (Patients on treatment with oral hypoglycaemic agents or insulin or with fasting plasma glucose ≥126 mg/dl, confirmed on a second occasion, or 2 hour post-challenge glucose (after 75 g OGTT) ≥200 mg/dl) irrespective of diabetes treatment;

• Need for statin treatment:

◦ Patients already receiving statin therapy irrespective of their actual LDL cholesterol and total cholesterol levels or

◦ Patients not currently on statin treatment with LDL-cholesterol levels ≥100 mg/dL (2.59 mmol/L) persisting after three months of dietary advise (Threshold levels for the initiation of statin treatment according to Third Joint Task Force of European and other Societies on Cardiovascular Disease Prevention in Clinical Practice [25]);

• Ability and willingness to comply with all study requirements.

#### Exclusion criteria

• Previous major vascular events (non-fatal myocardial infarction, non-fatal stroke, angina, transient ischemic attack, revascularization procedures, peripheral vascular disease);

• Poor metabolic control (HbA_1c _> 14.0%);

• Any condition requiring elective treatment with aspirin;

• Contraindications to aspirin (history of aspirin allergy, bleeding tendency, gastrointestinal haemorrhage or peptic ulcer within six months, active hepatic disease, asthma, renal failure, use of anticoagulant therapy.

• Contraindications to simvastatin (history of allergy, chronic liver disease or abnormal liver function [i.e. ALT or AST ≥1.5 times the upper limit of normal], severe renal disease or evidence of renal impairment [i.e. creatinine >2 mg/dl], concomitant use of potent inhibitor of CYP3A4.

• Chronic use of non-steroidal anti-inflammatory drugs;

• Presence of any life-threatening condition;

• Child-bearing potential (pre-menopausal women not using reliable contraception);

• History of active substance or alcohol abuse within the last year.

### Participating centres

The study will be conducted by diabetes specialists and general practitioners with a long experience of participation in clinical trials.

### Study treatments

All randomized patients will be instructed to take one or two tablets daily; in particular, if the patient will be assigned to "aspirin+simvastatin arm", he will be instructed to take one tablet containing simvastatin (one 20 mg tablet every evening), and the other containing aspirin. Otherwise, the patient will be instructed to take one tablet containing simvastatin.

Study drugs will be prescribed by the investigator.

An initial dose of 20 mg/daily of simvastatin will be prescribed to patients not previously on treatment with statins.

The dose of simvastatin can be increased to 40 mg/day at month 3 in those patients whose plasma LDL cholesterol remains ≥100 mg/dL (2.59 mmol/L).

Patients already on statins treatment at study entry will be shifted to the equivalent dose of simvastatin without the need for a drug-free period.

Changes in simvastatin doses are allowed throughout the study based on clinical judgment.

If target LDL cholesterol levels will not be reached with 40 mg/daily, clinicians may modify therapeutic strategies (changes of statin, addition of other drugs such as ezetimibe, fenofibrate, nicotinic acid, omega-3 fatty acid).

Treatment will be centrally assigned on telephone, after verification of the correctness of inclusion and exclusion criteria. A separate computer-generated randomization table will be produced for each physician/diabetes centre in random permuted blocks. Each randomized patient will receive a unique subject identification number.

### Sample size estimates

The sample size calculation is based on the cardiovascular event rate occurred in over 1000 diabetic patients without evidence of cardiovascular disease enrolled in the PPP trial [10]. This study was conducted with the participation of both General Practitioners and Diabetes Clinics in Italy, thus closely reflecting the setting of care and the context in which the ACCEPT-D trial will be conducted. In the PPP trial the cumulative incidence of major cardiovascular events (cardiovascular death, myocardial infarction, stroke, transient ischemic attack, peripheral vascular disease, revascularization procedures), after a median follow-up of 4 years, was of 11.5% in subjects not assigned to aspirin treatment (incidence rate of 2.88% per annum). Assuming that treatment with statins can reduce such an event rate by approximately one quarter (based on the results of the meta-analysis of primary prevention studies in patients with diabetes [26]), we can expect an incidence rate of major cardiovascular events of about 2.1–2.2% per annum. Since the primary cumulative end-point of the study also includes hospitalizations for major vascular causes, sample size calculations for the proposed trial are based on a conservative estimated 2.5% annual rate of major vascular events.

Aspirin has been shown to reduce major cardiovascular events by about one quarter in a wide range of high risk populations [27,28]. The sample size is thus estimated to detect with a statistical power of 90% (α = 0.05) a reduction in the risk of major cardiovascular events of 25% (HR = 0.75).

Given these assumptions, a total of 515 first events are needed for the primary efficacy analysis. Therefore, the total number of subjects to be enrolled is of 4.700 patients, to be followed up for five years. From the PPP trial [29], it is possible to foresee a trial discontinuation rate of 10%. Therefore, a total of 5.170 patients will be enrolled (2.585 in each treatment arm). A total of 515 events will also ensure an adequate statistical power (i.e. 80%) to detect a smaller reduction in the risk of major events (i.e. HR = 0.78).

### Statistical analysis

All the efficacy analyses will be based on the intent to treat population, consisting of all randomized patients.

Incidence rates (aspirin vs. no aspirin) will be estimated using Kaplan-Meier survival curves that will be compared using logrank analysis. Additionally, treatment efficacy will be assessed by multivariate analyses using Cox's regression model.

Other secondary analyses will include the evaluation of efficacy of aspirin on all secondary end-points. Aspirin efficacy will also be tested in the following different circumstances:

- Men vs. women;

- Age ≥65 vs. <65 years;

- Type of diabetes;

- Metabolic control (quartiles of HbA_1c_);

- Baseline cholesterol levels (quartiles of total and LDL-cholesterol levels);

- Presence of microvascular complications (retinopathy, nephropathy);

- Treatment with other cardiovascular drugs (in particular β-blockers, ACE-inhibitors, Angiotensin Receptor Blockers).

The Mantel-Haenszel procedure will be applied to test for the linearity of effects across subgroups, while the chi-square test will be applied to test for heterogeneity of effects among the subgroups.

#### Interim analyses

During the study one interim analysis will be performed after the achievement of a number of events equal to 50% of the planned number. Data will be analyzed with the double scope of verifying the correctness of the assumptions made for sample size estimation regarding the primary end-point event rate (this information will be used to provide guidance about the duration of follow-up), and to perform a formal efficacy analysis.

The Steering Committee can decide whether to end or modify the study if the comparisons in the study have provided "proof beyond reasonable doubt" (Appropriate criteria of proof beyond reasonable doubt cannot be specified precisely, but in general a difference of at least 3 standard deviations in an interim analysis of a major endpoint would be needed to justify halting, or modifying, such a study prematurely [30]) that for all patients the treatment is clearly indicated or clearly contraindicated in terms of a net difference in incidence of major end-point, or there will be evidence from other relevant trials or meta-analyses that might reasonably be expected to influence materially the patient management of many clinicians who are already aware of the other main trial results.

### Procedures

#### Visit schedule

After randomization, patients will be seen after three months, after six months, and every six months thereafter, to check tolerance and compliance to the trial treatments and to reassess the presence and level of cardiovascular risk factors and recording of outcome events.

All the patients should continue the protocol visit schedule even in case of treatment interruption or after the occurrence of one of the study events.

#### Data collection

For each randomized patient a minimum set of information will be collected at study entry. This will include clinical data regarding diabetes, cardiovascular risk factors, and selected concomitant medications. Information on laboratory parameters will also be collected, including HbA_1c_, lipid profile, serum creatinine, albumin-creatinine ratio, and liver enzymes. Investigators will be requested to report the value of the last measurement performed. If the last HbA_1c _measurement was performed more than 3 months before randomization and all the other measurements were performed more than 6 months before randomization, the measurement should be performed again, according to the standards of medical care for patients at high cardiovascular risk. Laboratory data will be abstracted from clinical records by the participating physicians and reported in ad hoc forms together with their reference ranges.

The same set of information will be collected at 6-month intervals, with the exception of albumin-creatinine ratio that will be collected annually (Table 1).

**Table 1:** Overview of the visits and measurements

	Follow-up			
Visit Number	1	2	3–4–5...	Final visit
Month	0	3	6-month intervals	
Inclusion/Exclusion criteria	x			
Informed Consent	x			
Medical history	x			
Concomitant Medication	x	x	x	x
Weight	x		x	x
Height	x			
Waist circumference	x		x	x
Blood pressure/Heart rate	x		x	x
HbA_1c_	x		x	x
AST, ALT	x		x	x
Creatinine	x		x	x
Microlbuminuria	x		x	x
Albumin/creatinine ratio	x		x	x
Total cholesterol, HDL cholesterol, Triglycerides	x	x	x	x
Adverse events		x	x	x
Compliance		x	x	x
End-point verification		x	x	x

The occurrence of any clinical event included in the end-points will be notified. An independent ad hoc committee of expert clinicians, identified by the Steering Committee, will validate all the clinical events.

Moreover, information on any adverse event, including side effects of study treatments, will be collected.

### Timing

The duration of the enrolment phase will be of 18 months.

The total trial duration is event-driven. A total of 515 subjects with a vascular event are required. A total duration of follow-up of 5 years after the first randomized patient is anticipated, if 5170 patients will be recruited and if the event rate will be of the same entity of that anticipated (incidence of 2.5% per annum).

If the number of patients enrolled or the event rate will be smaller, the duration of the follow-up will be prolonged to allow the achievement of 515 events.

### Ethical aspects

The study drugs are widely used in clinical practice and their risk profile is well known. Drugs safety will be closely monitored throughout the study.

Before starting the study, the protocol and/or other appropriate documents will be submitted to the Ethics Committee of the participating centres in accordance with the local legal requirements (see Additional file 1).

The study will be conducted in accordance with the EC guidelines for Good Clinical Practice and performed according to the revised Declaration of Helsinki.

The trial was registered March 2007 at the International Standard Randomized Controlled Trial Number Registration (ISRCTN48110081).

#### Patient information

A patient can be enrolled in the study only after a full discussion of all the aspects related to study design, implications for the patient, expected benefits and possible side effects, in line with the content of the patient information sheet, to be read and retained by the patient. The patient will be given time to consider fully the information given and will be encouraged to ask questions. If then he/she is willing to participate in the trial, he/she will be asked to sign a consent form.

#### Withdrawal from the study

A patient must be withdrawn from the study when judged necessary by the responsible physician or when the patient withdraws his/her informed consent. In these instances all outcomes should be assessed.

### Publication of the trial results

The trial results will be published by the members of the Steering Committee, on behalf of all ACCEPT-D Study Group. Before submission of any manuscript all local principal investigators will have the opportunity to comment on the manuscript.

## Abbreviations

ACCEPT-D- Aspirin and Simvastatin Combination for Cardiovascular Events Prevention Trial in Diabetes.

CV- Cardiovascular.

CVD- Cardiovascular Disease.

NO- Nitric oxide.

PAI-1- Plasminogen Activator Inhibitor-1.

PPP- Primary Prevention Project.

PROBE- Prospective Randomized Open Blinded Endpoint.

## Competing interests

GT is at present member of the Executive Committee of a trial sponsored by Bayer which is planned to test ASA in non diabetic moderate risk patients.

GDB, MS, GT, VE, AN declare that they received a grant for research from Bayer.

## Authors' contributions

GDB and MS contributed to the design of the study, drafted the manuscript, will be responsible for the recruitment and the follow-up of the patients enrolled, and will also participate in the analysis and interpretation of data.

AN and GT wrote the study protocol, have made substantial contributions to the conception and design of the study, obtained grant funding, will participate in the analysis and interpretation of data, and gave the final approval of the version to be published.

VE, AF, CBG, UV contributed in the design of the project, will participate in the interpretation of data, and gave the final approval of the version to be published.

## References

[B1] Haffner SM (2000). Coronary heart disease in patients with diabetes. N Engl J Med.

[B2] Miettinen H, Lehto S, Salomaa V, Mahonen M, Niemela M, Haffner SM, Pyorala K, Tuomilehto J (1998). Impact of diabetes on mortality after the first myocardial infarction. The FINMONICA Myocardial Infarction Register Study Group. Diabetes Care.

[B3] Haffner SM, Lehto S, Ronnemaa T, Pyorala K, Laakso M (1998). Mortality from coronary heart disease in subjects with type 2 diabetes and in nondiabetic subjects with and without prior myocardial infarction. N Engl J Med.

[B4] American Diabetes Association (2005). Standards of medical care in diabetes. Diabetes Care.

[B5] Giorda C, Nicolucci A (2003). Diabete mellito di tipo 2. Complicanze e rischio cardiovascolare in Italia.

[B6] Wald NJ, Law MR (2003). A strategy to reduce cardiovascular disease by more than 80%. BMJ.

[B7] Hippisley-Cox J, Coupland C (2005). Effect of combinations of drugs on all cause mortality in patients with ischaemic heart disease: nested case-control analysis. BMJ.

[B8] Evangelista V, Totani L, Rotondo S, Lorenzet R, Tognoni G, De Berardis G, Nicolucci A (2005). Prevention of cardiovascular disease in type-2 diabetes: how to improve the clinical efficacy of aspirin. Thromb Haemost.

[B9] Antithrombotic Trialists' Collaboration (2002). Collaborative meta-analysis of randomised trials of antiplatelet therapy for prevention of death, myocardial infarction, and stroke in high risk patients. BMJ.

[B10] Sacco M, Pellegrini F, Roncaglioni MC, Avanzini F, Tognoni G, Nicolucci A, PPP Collaborative Group (2003). Primary prevention of cardiovascular events with low-dose aspirin and vitamin E in type 2 diabetic patients: results of the Primary Prevention Project (PPP) trial. Diabetes Care.

[B11] Ridker PM, Cook NR, Lee IM, Gordon D, Gaziano JM, Manson JE, Hennekens CH, Buring JE (2005). A randomized trial of low-dose aspirin in the primary prevention of cardiovascular disease in women. N Engl J Med.

[B12] Sciulli MG, Renda G, Capone ML, Tacconelli S, Ricciotti E, Manarini S, Evangelista V, Rebuzzi A, Patrignani P (2006). Heterogeneity in the suppression of platelet cycloxigenase-1 activity by aspirin in coronary heart disease. Clin Pharmacol Ther.

[B13] Eikelboom JW, Hirsh J, Weitz JI, Johnston M, Yi Q, Yusuf S (2002). Aspirin-resistant thromboxane biosynthesis and the risk of myocardial infarction, stroke, or cardiovascular death in patients at high risk for cardiovascular events. Circulation.

[B14] Halushka MK, Halushka PV (2002). Why are some individuals resistant to the cardioprotective effects of aspirin? Could it be thromboxane A2?. Circulation.

[B15] Ridker PM (2003). Clinical application of C-reactive protein for cardiovascular disease detection and prevention. Circulation.

[B16] Steiner M, Reinhardt KM, Krammer B, Ernst B, Blann AD (1994). Increased levels of soluble adhesion molecules in type 2 (non-insulin dependent) diabetes mellitus are independent of glycaemic control. Thromb Haemost.

[B17] Otsuki M, Hashimoto K, Morimoto Y, Kishimoto T, Kasayama S (1997). Circulating vascular cell adhesion molecule-1 (VCAM-1) in atherosclerotic NIDDM patients. Diabetes.

[B18] Vinik AI, Erbas T, Park TS, Nolan R, Pittenger GL (2001). Platelet dysfunction in type 2 diabetes. Diabetes Care.

[B19] Friend M, Vucenik I, Miller M (2003). Research pointers: Platelet responsiveness to aspirin in patients with hyperlipidaemia. BMJ.

[B20] Albert MA, Danielson E, Rifai N, Ridker PM, PRINCE Investigators (2001). Effect of statin therapy on C-reactive protein levels: the pravastatin inflammation/CRP evaluation (PRINCE): a randomized trial and cohort study. JAMA.

[B21] Tan KC, Chow WS, Tam SC, Ai VH, Lam CH, Lam KS (2002). Atorvastatin lowers C-reactive protein and improves endothelium-dependent vasodilation in type 2 diabetes mellitus. J Clin Endocrinol Metab.

[B22] Liao JK (2002). Beyond lipid lowering: the role of statins in vascular protection. Int J Cardiol.

[B23] McFarlane SI, Muniyappa R, Francisco R, Sowers JR (2002). Clinical review 145: Pleiotropic effects of statins: lipid reduction and beyond. J Clin Endocrinol Metab.

[B24] Hansson L, Hedner T, Dahlof B (1992). Prospective randomized open blinded end-point (PROBE) Study. A novel desgin for intervention trials. Prospective Randomized Open Blinden End-Point. Blood Press.

[B25] National Cholesterol Education Program (NCEP) Expert Panel on Detection, Evaluation, and Treatment of High Blood Cholesterol in Adults (Adult Treatment Panel III) (2002). Third Report of the National Cholesterol Education Program (NCEP) Expert Panel on Detection, Evaluation, and Treatment of High Blood Cholesterol in Adults (Adult Treatment Panel III) final report. Circulation.

[B26] Vijan S, Hayward RA, American College of Physicians (2004). Pharmacologic lipid-lowering therapy in type 2 diabetes mellitus: background paper for the American College of Physicians. Ann Intern Med.

[B27] Antiplatelet Trialists' Collaboration (1994). Collaborative overview of randomised trials of antiplatelet therapy – I: prevention of death, myocardial infarction, and stroke by prolonged antiplatelet therapy in various categories of patients. BMJ.

[B28] Heart Protection Study Collaborative Group (2002). MRC/BHF Heart Protection Study of cholesterol lowering with simvastatin in 20.536 high-risk individuals: a randomised placebo-controlled trial. Lancet.

[B29] de Gaetano G, Collaborative Group of the Primary Prevention Project (2001). Low-dose aspirin and vitamin E in people at cardiovascular risk: a randomised trial in general practice. Collaborative Group of the Primary Prevention Project. Lancet.

[B30] Peto R, Pike MC, Armitage P, Breslow NE, Cox DR, Howard SV, Mantel N, McPherson K, Peto J, Smith PG (1977). Design and analysis of randomized clinical trials requiring prolonged observation of each patient. II. Analysis and examples. Br J Cancer.

